# Supplementary material for: COVID-19 Infection May Drive EC-like Myofibroblasts towards Myofibroblasts to Contribute to Pulmonary Fibrosis
Source: Int J Mol Sci. 2023 Jul 15;24(14):11500. doi: 10.3390/ijms241411500 (PMC10380846; doi:10.3390/ijms241411500)
Supplement: Supplementary file 1 [file ijms-24-11500-s001.zip › ijms-2463142-supplementary.pdf]

Supplemental Information

Covid-19 infection may drive EC-like myofibroblasts towards myofibroblast to contribute pulmonary fibrosis

Xiuju Wu [1,†], Daoqin Zhang [2,†], Kristina I. Boström [1,3,*] and Yucheng Yao [1,*]

[1] Division of Cardiology, David Geffen School of Medicine at UCLA, Los Angeles, CA 90095-1679, U.S.A.
[2] Department of Pediatrics, Stanford University, Stanford, CA 94305, U.S.A.
[3] The Molecular Biology Institute at UCLA, Los Angeles, CA 90095-1570, U.S.A.
† These authors contributed equally to the work.
* Correspondence: yyao@mednet.ucla.edu (Y.Y.); kbostrom@mednet.ucla.edu (K.I.B.); Tel: 310-825-3239, Fax: 310-206-8553

Supplemental Figure 1 and description

Supplemental Figure 2 and description

Supplemental Figure 3 and description

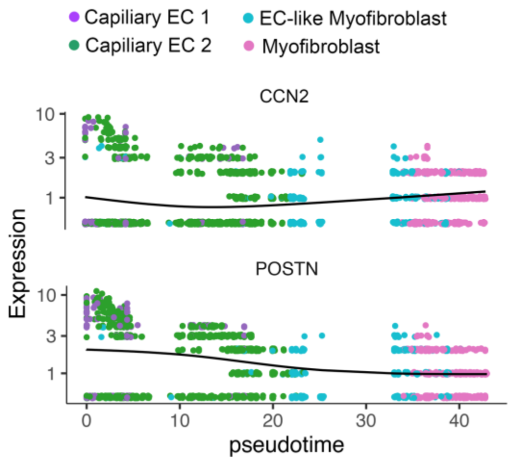

Supplemental Figure S1: The expression of CCN2 and POSTN along single cell trajectories.

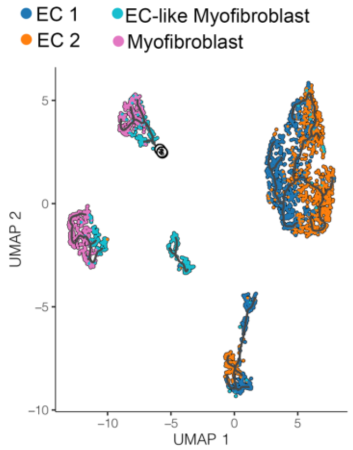

Supplemental Figure S2: Pseudotemporal trajectories of the cell clusters, which projected no connection between EC 1 or 2 and EC-like myofibroblast or myofibroblast.

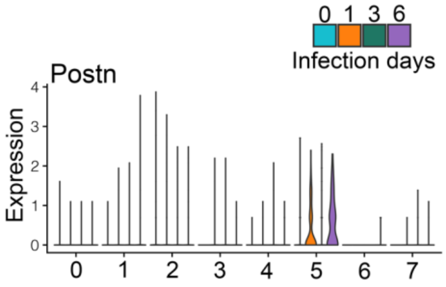

Supplemental Figure S3: The expression of Postn in EC-like myofibroblasts from mouse lungs with influenza A infection.
